# RiboFSM: Frequent subgraph mining for the discovery of RNA structures and interactions

**DOI:** 10.1186/1471-2105-15-S13-S2

**Published:** 2014-11-13

**Authors:** Alex R Gawronski, Marcel Turcotte

**Affiliations:** 1 School of Electrical Engineering and Computer Science, University of Ottawa, 75 Laurier Ave E, K1N 6N5, Ottawa, Canada; 2 School of Computing Science, Simon Fraser University, 8888 University Dr, V5A 1S6 Burnaby, Canada

**Keywords:** RNA, graph mining, dual graphs

## Abstract

Frequent subgraph mining is a useful method for extracting meaningful patterns from a set of graphs or a single large graph. Here, the graph represents all possible RNA structures and interactions. Patterns that are significantly more frequent in this graph over a random graph are extracted. We hypothesize that these patterns are most likely to represent biological mechanisms. The graph representation used is a directed dual graph, extended to handle intermolecular interactions. The graph is sampled for subgraphs, which are labeled using a canonical labeling method and counted. The resulting patterns are compared to those created from a randomized dataset and scored. The algorithm was applied to the mitochondrial genome of the kinetoplastid species *Trypanosoma brucei*, which has a unique RNA editing mechanism. The most significant patterns contain two stem-loops, indicative of gRNA, and represent interactions of these structures with target mRNA.

## Introduction

In most organisms the process of protein synthesis is well understood. Deoxyribonucleic acid (DNA) is transcribed into messenger ribonucleic acids (mRNA), which are then translated into polypeptides that fold to create proteins. However there are a few families of organisms where the process deviates from the norm. One such family is the *Kinetoplastida *in the kingdom *Excavata*. The mRNA produced from the mitochondrial DNA of this family cannot be directly translated into protein but must be prepared by a process called RNA editing. This process is mediated by short RNA molecules called guide RNA (gRNA) [1]. Another closely related family, Diplonema, also has a unique editing system in its mitochondria. In this case the genes are fragmented into "modules" which are transcribed separately and then assembled by an unknown mechanism [2].

Little work has been done in the development of computational methods for discovering gRNA. The existing methods suffer from poor precision and dependence on experimental transcript data [3,4]. Even less has been done for Diplonema. The current research has only shown that known cis-splicing mechanisms are not present and suggests that RNA guides or proteins mediate the process [2]. A computational approach was used in one study but again suffered from a lack of precision, generating millions of candidate structures [5].

The objective of this new methodology is to discover RNA interactions occurring in known or novel RNA-mediated mechanisms. These mechanisms involve RNA with a specific secondary structure formed by complementary sequences within the molecule. Depending on the location of these stems different substructures can be created. There are four basic substructures: stem-loops, interior loops, bulges and pseudoknots. These RNA also contain complementary sequences to other RNA, such as target mRNA, to allow them to form a quaternary structure. If all possible stems between a set of molecules were known, any RNA mechanism would be a subset of those stems. The problem then becomes finding that correct subset of stems. However the number of possible stems is very large, and the number of combinations of these stems is enormous.

The first challenge is determining how to represent the data. The information of interest is the position of the complementary sequences, the length of these sequences, and the relative locations of these sequences. Since this data is many single units of information, interconnected by there relative locations, it is well suited for a graph representation. Furthermore, graphs are ideal for representing complex topologies, which in this context allows for representation of complex RNA structures and interactions. Graph representations for RNA structure have been used in other studies, usually using a planar tree graph representation [6]. In tree graphs, loops are collapsed into nodes and the stems forming the loop become edges. The main drawback with tree graphs is that pseudoknots cannot be represented. A proposed solution was to add additional edges between nodes that form pseudoknots [7] however this breaks down the tree representation and a special case must be made for matching these structures. Recently an extension of tree graphs was developed, call 3D tree graphs, for modeling tertiary structures [8].

**Figure 1:**
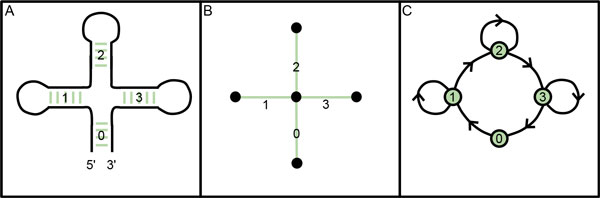
**Tree graph versus dual graph example**. Example RNA structure (A) with corresponding tree graph (B) and directed dual graph (C) [6].

An alternative solution is to use the reverse representation, that is, stems become nodes and loops become two edges. With this modification pseudoknots can be represented by three edges between two nodes. This type of graph is called a dual graph [6]. However some ambiguities may occur with this representation. The order of stems while going along the RNA strand can be modified but would still produce the same dual graph. This can be resolved by creating directed edges, creating the directed dual graph representation, which was used in this work [6]. This representation is more robust than tree graphs with no disadvantages, and its application to modeling the interactions between many RNA molecules has not been explored. An example of each graph representation is shown in Figure 1.

Recently this representation was used on a moderately large scale to produce the RNA as Graph (RAG) database [6,9]. This database stores dual graph representations of all known RNA motifs as well as their tree graph versions. The algorithm produced approximately 53,000 dual graphs up to a size of 9 nodes from individual RNA, which they classified as RNA-like and non-RNA-like. This is dwarfed by the search space of all possible RNA structures across multiple molecules, which is in the order of tens of millions of subgraphs.

Another graph representation using the reverse representation is a "stem graph" developed by Hamada et. al. [10]. This is a directed, labeled graph where the nodes represent candidate stems. The edges represent the relative locations of the stems and are labeled P (Parallel), N (Nested) or K (Pseudoknotted) to capture how stems are orientated. Like dual graphs, this representation can support pseudoknots because of this labeling technique. However this approach requires edges from each node to all the downstream nodes. This could cause scalability issues for complex or large structures.

A graph created from all complementary sequences will be largely noise. An accepted method for extracting useful data from graphs is called Frequent Subgraph Mining (FSM). FSM is the process of finding subgraphs in a graph with a frequency no lower than a specified threshold. In the single graph setting, the number of "embeddings" of a subgraph within the graph are counted to determine frequency. This presents unique challenges since many embeddings can be overlapping which could lead to the frequency not being downward closed. More specifically, the anti-monotonicity property would not hold, which declares that a subgraph can only be frequent is all of its subgraphs are frequent [11].

Many approaches solve this problem by creating an overlap graph [12,13], where a subgraph instance is a node and an edge exists between nodes if the instances overlap. The size of the maximum independent set (MIS) is determined and is used as the support value. This is costly since finding the MIS is a NP-complete problem [14] so generally it is approximated or alternative approaches are used. Fiedler and Borgelt [15] suggested a definition called harmful overlap support. This method still calculates the MIS, however it divides overlaps into two kinds, harmful and simple. The process is sped up by ignoring the simple overlaps and still maintains anti-monotonicity. Bringmann and Nijssen [16] defined a new support measure that does not rely on the calculation of MIS but instead on the number of unique nodes in the graph to which a node of the pattern is mapped. Therefore it is less expensive computationally than the other two methods, but still relatively costly.

Another challenge is determining whether two subgraphs have the same topology. This is called the subgraph isomorphism problem and it is also NP-complete [17]. Various approximations have been proposed including the use of canonical labeling. Canonical labeling allows for a "code" to be assigned to a subgraph that will be consistent even if the order of vertices and edges changes [12]. This is accepted as the fastest method for determining subgraph isomorphism [12,18,19]. There was no need to expand this method or develop a new method.

A difficulty of single-graph FSM is the often enormous size of the input graph and consequently the search space. Few algorithms attempt to search the entire search space and do not scale well, such as hSiGraM/vSiGraM [12]. Most approaches use heuristics or stochastic methods to find approximate solutions. Some examples are compression-based methods (SUBDUE [19]), pruning methods (GREW [13]) and sampling methods [20] in order of how well they scale, worst to best.

We present here a generalization of the directed dual graph for both intramolecular and intermolecular complementary regions, thus capturing secondary structure as well as interactions between RNA structures. We developed a new frequent subgraph mining method specifically for this type of representation and show that it can be effectively used to find *de novo*, biologically plausible structures. This is also the first application of FSM on dual graphs and of FSM for the discovery of RNA motifs and interactions in a single graph (including intermolecular) setting.

## Methods

### Finding complementary regions

The first step of the algorithm is to find all possible complementary regions between the input sequences. A complementary region of a sequence is another sequence where the nucleotides complement each other (A-U/T, C-G, G-U/T) and are in reverse order. The algorithm used to match the sequences is a naive method involving matching each position *i *in a sequence of size *n *with every other position *n-j*, with some heuristics. Firstly, since *i *and *j *are indexing the same sequence, comparing *i *= 1 to *j *= 2 is equivalent to *i *= 2 to *j *= 1. Therefore the heuristic of enforcing *j > i *is used to avoid finding duplicate matches. Secondly the maximal match is always used and the match positions are stored from the last iteration so *i *+ 1 is not matched to *j − *1. This avoids the creation of nested matches which would greatly increase the number of nodes without adding any more information. The matches must be of specified minimum size and can only contain a certain percent of G-U matches.

An alternative approach for filtering matches using stacking energy was also implemented. This is done by summing the stacking energies of each matching pair of nucleotides based on the previous matching pair. If mismatches are allowed, the mismatch energy is used for mismatching pairs. The energy values and procedure were derived from the Vienna package implementation [21]. Consequently every node created has an associated free energy in kcal/mol. This value can be used to filter nodes to reduce the size of the graph and improve the quality of the final structures. Shorter stems and stems with higher G-U pairs are naturally filtered in this way.

### Graph representation

The graph representation used is the directed dual graph [6]. In this representation every complementary region is represented as a node. Unpaired nucleotides are represented as edges which are directed in the 5' to 3' direction. The graph is also not necessarily fully connected since edges are only created between nodes on the same molecule. This representation was adapted to handle intermolecular interactions by adding the notion of intermolecular nodes. Edges are only created between nodes on the same molecule. This allows all possible interactions to be captured without the creation of excess edges for each node.

**Figure 2:**
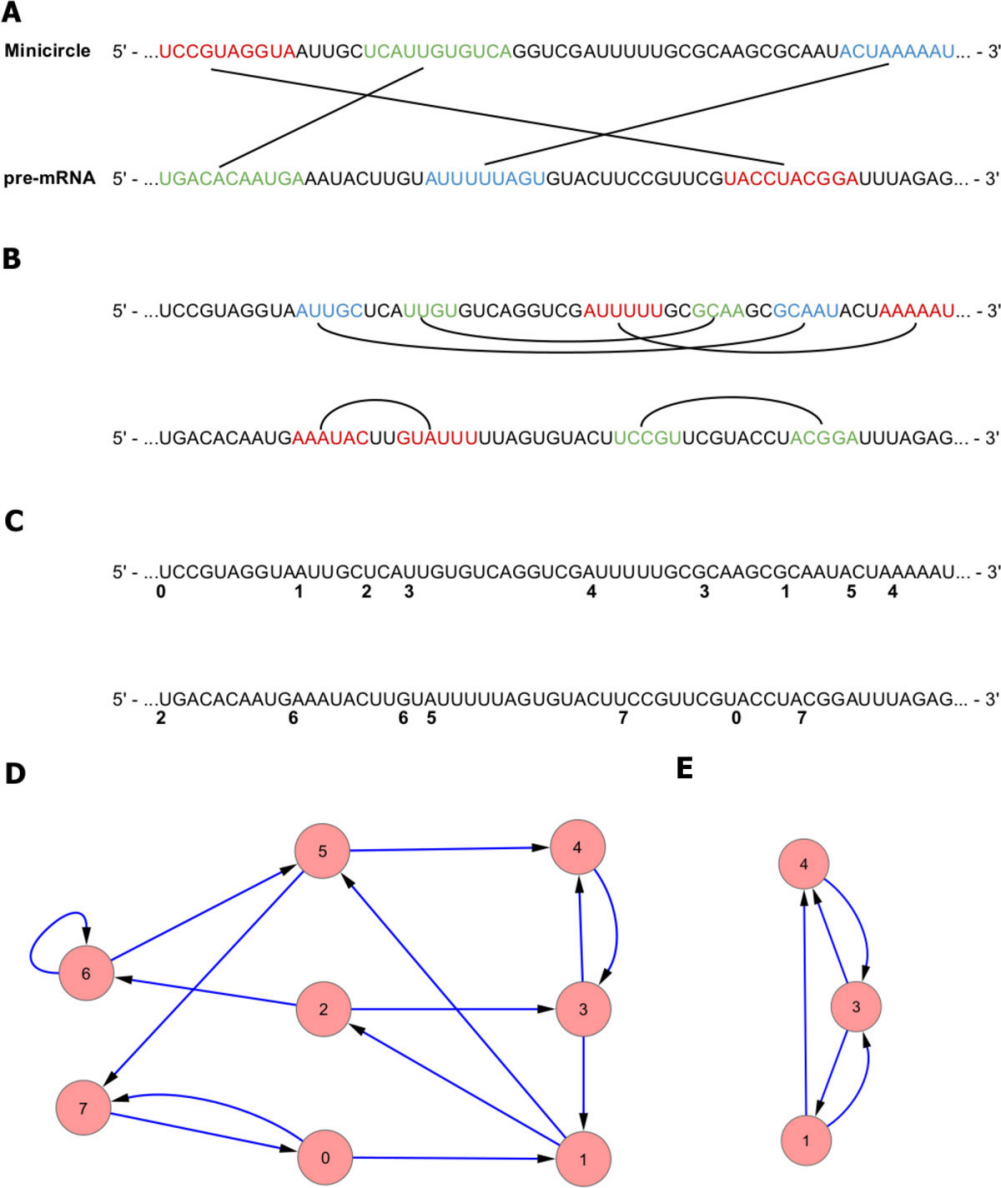
**Example of graph building given two input sequences**. Example of graph building given two input sequences. All intermolecular (A) and intramolecular (B) complementary regions are discovered and enumerated (C). Traversing the sequence using the priority queue, the nodes corresponding to each region can be created to form the final graph (D). An example of a three node subgraph being sampled from a dual graph (E).

Every node can have at most two predecessors and two successors. The nodes at the 5' and 3' ends of the sequences will have one fewer. This leads to the relation *|E| *= 2*|V | − *1, which indicates the graph is (2,1)-sparse. The sparseness of this representation justifies the use of an adjacency list rather than a matrix. However since the edges are always between adjacent nodes the data structure can be simplified to a list of nodes. Additionally every node has a reference to a "sister node" elsewhere in the list. This allows for both sequences in a match to be treated as one node while still maintaining the simplicity of the list data structure. In other words, if you traversed the sequence from 5' to 3', every node will be visited exactly twice, and that path would create the graph (Figure 2).

When a complementary region is found, two nodes are created. One node is for the sequence at the *ith *position and the other is the sister node with the sequence at the *jth *position. The first node is added to the master list of nodes and the sister node is added to a priority queue. The priority queue ensures the nodes are sorted based on position. If the priority queue is not empty it is checked for nodes that have an index matching the current position. If such a node exists it is popped out of the queue and added to the master list (Algorithm 1). The procedure builds the graph in *O*(*nlogn*) where *n *is the number of complementary regions, due to sorting occurring in the queue.

**Algorithm 1 **Pseudo-code for building the graph.

1: *max *= last position

2: *queue *= new PriorityQueue

3: *graph *= new Graph

4: **for all ***Sequences *: *S ***do**

5:    **for ***i = S.start; i ≤ S.end; i + + ***do**

6:       **if **queue not empty **then**

7:          *sisN ode = queue*.peek()

8:          **while ***sisN ode*.location = i **do**

9:             *graph*.addNode(*sisN ode*)

10:           *sisN ode = queue*.peek()

11:         **end while**

12:       **end if**

13:       **for ***j *= *max*; *j ≥ i*; *j − − ***do**

14:         **if **Not nested of last match **then**

15:           **if **match(i,j) **then**

16:             create *node*

17:             create *sisN ode*

18:             *graph*.addNode(*node*)

19:             *queue*.add(*sisN ode*)

20:           **end if**

21:         **end if**

22:       **end for**

23:    **end for**

24: **end for**

### Sampling of subgraphs

The created graph now becomes the search space for frequent subgraph mining. Since edges are the unpaired nucleotides between two adjacent nodes, two nodes that are not adjacent would not have an edge connecting them but still form a valid subgraph. This makes the use of a pattern-growth method for searching the graph insufficient since many potential graphs will be missed. Therefore it is necessary to sample sets of nodes, which changes the edges between the nodes. For example, if the nodes 1, 3 and 4 are selected from the graph in Figure 2 D, it would produce the subgraph in Figure 2 E. Note that the edges (1, 2) (2, 3) and (1, 5) (5, 4) become edges (1, 3) and (1, 4) respectively.

This causes a unique challenge that cannot be solved with common FSM algorithms. This is because formally the subgraphs produced are actually graph minors, which is a graph produced by contracting edges of the original graph. Since the inspiration for this work was drawn from mining traditional subgraphs, we will adopt the term "subgraph" for the graph minors produced. Consequently the search space is much larger than the set of all true subgraphs. The search space is large enough that a deterministic search of all subgraphs is infeasible. For this reason it was decided that a sampling approach would be used with the assumption that if the sample size is large enough the proportions of frequent subgraphs in the sample would be equal to that of the entire graph.

The sampling method used, k-nearest neighbor, uses a *k *parameter rather than a sample size parameter. A random node is selected in the graph and all overlapping nodes are flagged as "unavailable". The algorithm then adds (*k − *1)*/*2 nodes in both the 5' and 3' direction until a subgraph of size k is created, if enough nodes are available. This method is preferred over the random method since it has a bias toward creating structures with elements close to each other which are more likely to occur in nature. Furthermore it draws out more intramolecular nodes, which are vastly outnumbered by intermolecular nodes. This method has an additional option to allow unrestricted overlap of intermolecular and intramolecular nodes, but not two nodes of the same type. This allows subgraphs to represent both states of a mechanism, which are when the molecules are separate and when they are bound.

### Canonical labeling

To determine the frequency of each subgraph, isomorphic graphs need to be grouped and counted. This is accomplished by labeling each subgraph based on topology, node labels and edge labels. The combination of these labels allows for the canonicalization of the subgraphs and greatly increases the speed of comparing graphs. In the general case for simple undirected graphs, this process is as difficult as the subgraph isomorphism problem which is NP-Complete. This is because for a given subgraph, all possible permutations of node/edge labels must be compared and the lexicographically largest or smallest label must be selected [12]. Due to the ordered nature of the directed dual graph representation, the nodes can only be arranged in one way and therefore only one label can exist. This makes it possible for the labeling process to be completed in linear time with respect to the size of the subgraph.

Each subgraph is labeled and compared to other already labeled subgraphs. At this point a unique label based on node IDs is checked for determining automorphism which ensure no duplicates are counted. Every time a unique subgraph is found a "pattern" is created which encapsulates the label information and stores instances of the pattern. Subsequent subgraphs are compared to existing patterns and either added or form new patterns. The combined labels are hashed for fast access and matching. Once all subgraphs are labeled the final output is the set of patterns.

All labels are sequences of digits or boolean values separated by pipe characters. The topology label is created by assigning indices to nodes as they are visited in the subgraph. If a sister node is reached the index of first instance is used. This method can capture any dual graph topology and will have a one-to-one mapping of labels to topologies. Match length and intermolecular/intramolecular labels are the values assigned to the nodes, in the order that the nodes are visited. The former describes how many nucleotides are in the complementary sequence and the latter whether the match is between sequences on the same molecule or different molecules. The length values are normalized to a discrete scale, 0 to 5 by default (Table 1).

**Table 1:** Labels corresponding to the graph in Figure 1

Property	Label
Topology	0|1|1|2|2|3|3|0
Match Length	4|5|4|5
Inter/Intra	Intra|Intra|Intra|Intra

### Determining statistically significant patterns

Frequencies of patterns on their own are not very useful for determining biologically relevant patterns. Some patterns are far more likely to occur over others by chance. For example a pattern containing one node with the minimal match length will always be most frequent, but is not very useful for further analysis. To solve this problem the algorithm runs twice, once with the real data and again with the data randomize. The randomization was done using a Markov chain of order 1 to randomize the input sequences while maintaining nucleotide and dinucleotide frequencies. The size and number of molecules were also maintained. This produces two sets of patterns which can then be compared to determine patterns that are significantly more frequent in the biological data. Both the observed graph and background graph may have millions of nodes, making it infeasible to have multiple randomized background graphs.

Each pattern in each set has a number of unique embeddings. The first step is to determine the proportion of embeddings (*p*) associated with each pattern by dividing the frequency (*f*) over all embeddings (*N*) (Equation 1). The total number of embeddings is equal to the number of valid subgraphs sampled from the entire graph. For the proportions of the sample to reflect the real proportions, the number of subgraphs selected in the sample must be large. As the number of subgraphs sampled approaches the total number of possible subgraphs, the hypothetical proportions approach the real proportions of the graph. A benefit of using proportions over raw frequency values is that they are relative to the total number of subgraphs, which will vary between the two sets.

The next step is to find all the differences between the proportions for each pattern. Due to the large number of embeddings and patterns, the proportions are very small. Taking the differences of these numbers would yield another set of very small numbers. Furthermore there would be a bias towards patterns with large numbers of embeddings which can have large numerical differences even if they are relatively close. For this reason the ratio between proportions are used. This ratio is calculated by dividing the observed proportion (*p_obs_*) by the expected proportion (*p_exp_*) (Equation 1).

To avoid divide-by-zero errors, if the expected frequency is zero then the frequency is set to one. Since the expected frequency cannot be predicted and assuming the value is one skews the distribution, these values are not used in further calculations. However they are retained to estimate the p-value for rare patterns after the distribution is created. Conversely if the observed frequency is zero, then the pattern is ignored also because any assumption would skew the distribution. Therefore the ratios used for the final step are only those that correspond to patterns that exist in both sets.

(1) $$\begin{array}{c} p_{obs}=\frac{f_{obs}}{N_{obs}}\\ p_{exp}=\frac{f_{exp}}{N_{exp}}\\ lpr=log\left(\frac{p_{obs}}{p_{exp}}\right) \end{array}$$

**Figure 3:**
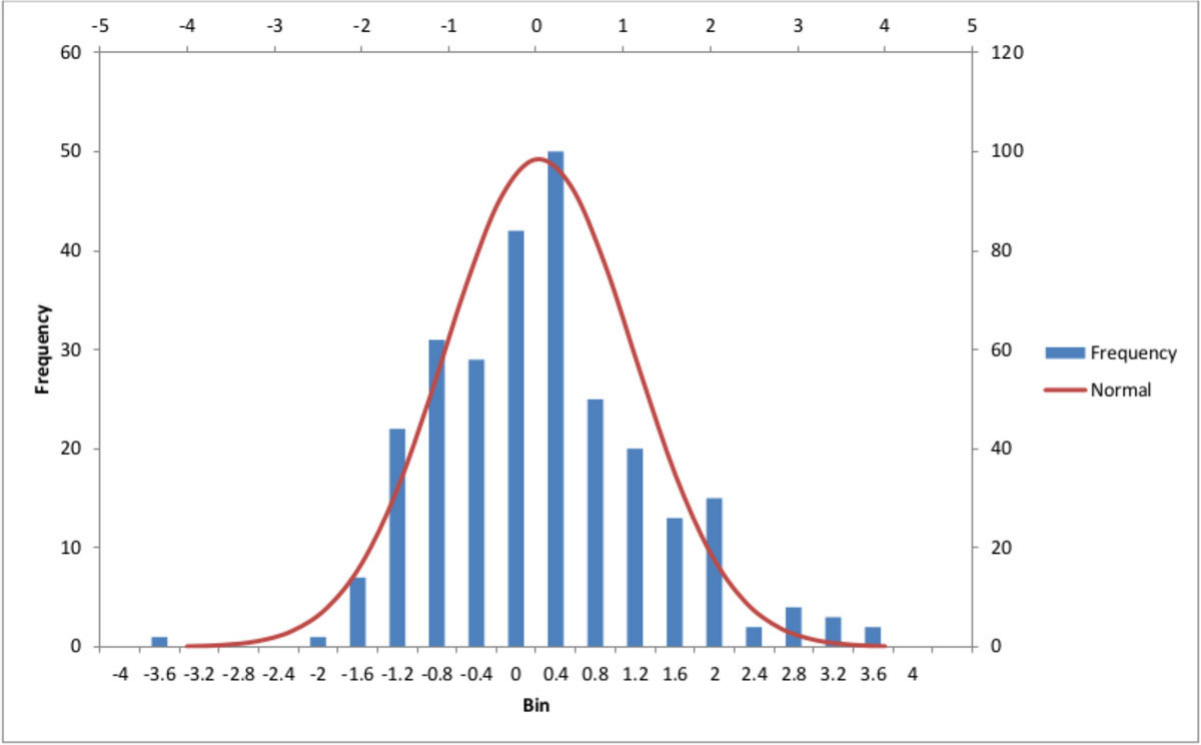
**Histogram of log ratios**. Histogram of log ratios of the proportions of subgraphs for patterns produced from the synthetic data set.

The final step is to examine the resulting set of ratios. The distribution of the ratios themselves lay between one and zero where most observations are close to one and drop exponentially when moving towards zero. However taking the log of the ratio produces an almost normal distribution (Figure 3). As would be expected there is a skew towards the positive end since they correspond to patterns found in higher frequency in the observed results. How close the distribution is to the normal distribution was quantified using an Anderson-Darling test for normality [22]. The test yielded a p-value between 0.03 and 0.05, which means the difference between the distributions is just significant enough to reject the null hypothesis. However the Anderson-Darling test is very sensitive and therefore it was decided that the distribution is sufficiently close to normal to make the assumption that it is normal. The consequence of this assumption is that some p-values may be smaller than those produced from the actual distribution and subsequently overestimate the significance of certain patterns.

The mean and standard deviation of these log ratios are calculated and used to create a normal distribution. From this distribution the p-value is calculated for each pattern's ratio by determining one minus the cumulative probability. Patterns are then ranked by p-value and those with the best p-value represent potential biological mechanisms. The construction of the normal distribution and operations on the distribution were implemented using Apache Math Commons version 3.2.

## Experimental setup

Two kinds of data sets were used for evaluation. The first is a controlled synthetic data set and the second is a real data set. Synthetic data is produced by generating random sequences and inserting positive examples. Other examples can be inserted to create various levels of noise. The real data set is from biological data with known examples that can be verified. If the algorithm can perform well for these two cases, where the performance can be empirically determined, then the algorithm can be used with confidence on an unknown data set. A third data set, the mitochondrial genome of *Diplonema papillatum*, was also used as a data set with an unknown mechanism.

### Synthetic data

The synthetic data is based on the gRNA mechanism of *Trypanosoma brucei*. The input is split into two sequence types, minicircles and mRNA. 200 sequences are synthetic minicircles and 5 sequences are synthetic mRNA, each sequence being 800nt. The ratio of minicircle to mRNA as well as the length of each sequence was derived from real data. The true ratio of minicircle to mRNA is approximately 38:1 which is close to the used ratio of 40:1. Maxicircle mRNA transcripts are an average length of 737nt [3], which was rounded to 800nt for this data set. Minicircle lengths for *Trypanosoma brucei *are, on average, approximately 1Kb long [23], however 800nt was selected to have a balanced data set. The nucleotide composition was determined by counting nucleotide frequencies in the real data. The frequencies were determined by chromosome type and the approximate percent composition of each nucleotide was used to generate the synthetic sequences.

The model gRNA were made of three complementary sequences. Going from 5' to 3' these are an anchor sequence followed by two stem-loops. The anchor overlaps withe the first stem-loop as it does in nature [24]. The anchor is 12nt long, based on the maximum size provided in the work of Hajduk and Ochsenreiter [25]. Stem-loop stem sizes vary so a conservative size of 10 bp was chosen. The spacing between these elements was chosen to be 5nt. This produces a pattern with a label "0|1|1|2|2-inter|intra|intra", however the pattern label "0|1|0|2|2-intra|inter|intra" would also be acceptable if the stems were extended by chance.

An algorithm was then used to insert model gRNA into the sequences by modifying regions of the sequences. This algorithm identifies three regions of appropriate length and location. The first is stored and the reverse complement is inserted into one of the mRNA, creating the anchor. The second and third are also converted into reverse complements and inserted downstream to created the stem-loops. Four gRNA were inserted into each minicircle which is the median number of gRNA genes per minicircle, with the range being between three and five gRNA [23].

### *Trypanosoma brucei *data

The second data set used to test the algorithm was a real biological dataset from the public KISS database [3]. The complete data has minicircle sequences and mRNA transcripts, both edited and unedited. Since the unedited mRNA is included in the edited mRNA, only the edited mRNA was used. This also reduces the number of false positives due to anchors being found in unedited regions. The data also includes mRNA from normal genes that do not undergo editing, which were removed. In the context of RNA editing, it is often simple to determine which mRNA undergoes editing by comparing the sequence to experimentally determined mature mRNA or polypeptide sequences. The final data set includes 455 minicircles and 12 mRNA sequences. The approximate total length was 358,000nt for the minicircle sequences and 8,800nt for the mRNA sequences.

### *Diplonema papillatum *data

This data set was used to test the algorithm in a case where the mechanism is unknown. The data was taken from the work of Kiethega et. al. [5]. It includes the complete mitochondrial genome as well as expressed sequence tags (EST) which were used as the first input where ppRNA are expected to be found. This portion of the data contains approximately 6,000 sequences. The second input are the 9 modules of the cytochrome oxidase subunit 1 gene, since this is the only gene the authors have sequenced so far. The total size of the data set is 3,450,000nt for the first input and 1600nt for the second. All ESTs with unknown sequences (only 'N' characters) were removed and areas with low complexity were masked with 'N' characters. This brought down the size of the data set to 2,816,000nt. This is still significantly larger than the other data sets so it was also useful for testing the scalability of the algorithm.

### Metrics

For metrics, precision and recall were used, generated from a contingency table. Since the data is imbalanced (1:150 positive to negative example ratio), precision and recall are more effective than accuracy and specificity as a metric of performance [26]. In the context of the experiments the examples are subgraph embeddings. The actual positive examples are the inserted or real gRNA. When the synthetic data set was used, the subgraphs must have a label matching the correct pattern label and have node locations that are within a specified distance from the actual positions. This distance was set to 5nt for all test since any extension beyond this would be unlikely to occur by chance.

The predicted positive examples are the embeddings corresponding to the patterns which meet p-value and frequency thresholds. Since a sampling technique is used the total number of real positive examples only includes those that were sampled and all others remain unclassified. This is unlike conventional data mining techniques where the complete set of positive and negative examples can be enumerated. This is an important factor to consider when interpreting the results.

The *Trypanosoma brucei *data set is from the KISS database which also stores all experimentally verified gRNA as well as gRNA predicted using a modified BLAST search. These matches are stored in General Feature Format (GFF), which were parsed to extract the locations of anchors where the gRNA binds to the pre-mRNA. Whenever a frequent subgraph is identified, it is checked for an intermolecular node. The start and end locations of this node with respect to each input sequence are extracted. These locations are then compared to those of the real locations from the GFF files. If a match is found, the subgraph is considered a true positive (or false negative). This method is not as stringent as the method used for synthetic data.

The objective score used to compare the final results is the F-score (Equation 2). This score is based on the harmonic mean of the precision and recall. The harmonic mean is different from the arithmetic mean because it is less sensitive to outliers. This is appropriate in this context since values closer to each other are preferred. For example, 1% precision and 100% recall would have a higher arithmetic mean than 50% precision and 20% recall, even though it is less meaningful. However the harmonic mean is higher in the latter than in the former. The F-score is also weighted (*β*), which allows for favoring precision over recall or vice versa. In these experiments the value is fixed to 1, since neither is preferred. The F-score is compared to the F-score of the baseline, calculated using the same number of positive and negative examples as the actual result but chosen at random.

(2) $$F_{\beta }score\;=\;\left(1\;+\;\beta^{2}\right)\;\times \;\frac{precision\;\times \;recall}{\left(\beta^{2}\;\times \;precision\right)\;+\;recall}$$

## Results and discussion

### Synthetic data

Analyses of each parameter was carried out and a test case of the best parameters was created (Table 2). This test case has 8 different combinations with 6 replicates per parameter combination. The average of the 6 replicates was used for comparison.

**Table 2:** Parameters selected for the final experiments.

Run	Length	% GU	Iterations	K	Genes	Norm	P-Value	Support
**1**	9	0	1000000	3	2	2	0.00001	10
**2**	10	0	1000000	3	2	2	0.00001	10
**3**	9	0	1000000	3	2	2	0.00001	20
**4**	10	0	1000000	3	2	2	0.00001	20
**5**	9	0	1000000	3	2	8	0.00001	10
**6**	10	0	1000000	3	2	8	0.00001	10
**7**	9	0	1000000	3	2	8	0.00001	20
**8**	10	0	1000000	3	2	8	0.00001	20

The results of this test case are summarized in Table 3. The initial results showed precision values significantly lower than the recall. For this reason the test case was run again with a more stringent p-value. The best score was for run number 4, with a F-score of 63.18%. The best single run was the third replicate of run number 2 which yielded a precision of 98.31% and recall of 79.48%. This run also had the maximum precision of all runs. The highest recall recorded was 97.57% from the third replicate of run 4. The specific replicates are not shown here but are provided in [27]. All tests performed much better than the random classifier (P = 6.29 *× *10*^−5^*), as determined by a paired t-test.

**Table 3:** Average F-scores across replicates for each combination of parameters from Table 2 for the synthetic data set.

	Observed			Baseline			
**Run**	**Precision**	**Recall**	**F-Score**	**Precision**	**Recall**	**F-Score**	**F-Score Diff**

**1**	46.81%	67.77%	55.38%	5.13%	6.52%	5.74%	**49.63%**

**2**	42.72%	52.95%	47.29%	16.65%	16.03%	16.33%	30.95%

**3**	38.80%	57.36%	46.29%	4.70%	7.79%	5.86%	40.43%

**4**	**51.21%**	**82.46%**	**63.18%**	15.74%	23.76%	18.94%	44.24%

**5**	19.71%	63.73%	30.11%	5.44%	16.39%	8.17%	21.94%

**6**	25.38%	82.33%	38.79%	15.33%	53.32%	23.82%	14.98%

**7**	17.84%	45.99%	25.71%	5.70%	14.14%	8.12%	17.59%

**8**	27.64%	76.46%	40.60%	16.39%	45.38%	24.08%	16.52%

**Avg**.	33.76%	66.13%	43.42%	10.64%	22.92%	13.88%	29.53%

Bold results are the best for the given metric.

The baseline performance varies between runs with some runs having higher precision and recall than others. This may be an indicator that a specific run is only better than others because it should be better simply by chance. To objectively compare the various runs, the results should be corrected depending on the corresponding baseline. To accomplish this, the F-score of the baseline results are subtracted from the F-score of the observed results. Considering this, the best result is run number 1, with a 5% higher corrected F-score than run number 4.

The best result produces exactly one significant pattern which contains nearly all of the inserted structures (Table 4). This is the pattern containing an anchor and two stem-loops where the anchor starts just before the first stem-loop. The other runs for this set of parameters also included this pattern, as well as a similar pattern that have the anchor incorrectly downstream of the two stem-loops. The average p-value for all of the runs was 8.04 *× *10*^−7^*. The false negatives are most likely the other correct pattern of |0|1|0|2|2|, where the anchor is entirely within the hairpin. The only way for this result to be better would be for the second correct pattern to be the second result. The other two results for run 2 were similar, with one or two patterns with high frequency of correct embeddings.

**Table 4:** The pattern result of the best run (run 2.3).

P-value	Frequency	Label
9.40E-7	532	0|0|0|-|0|1|1|2|2|-|inter|intra|intra|

### Trypanosoma brucei data

Like the synthetic data set, a test case of the best parameters was created (Table 5). This test case has 8 different combinations with 6 replicates per parameter combination. The average of the replicates is used for comparison.

**Table 5:** Parameters selected for the final experiments.

Run	Length	% GU	Iterations	K	Genes	**Norm**.	P-Value	Support
1	10	0.4	1000000	3	2	2	0.01	1
2	10	0.4	1000000	3	2	5	0.01	1
3	12	0.4	1000000	3	2	2	0.01	1
4	12	0.4	1000000	3	2	5	0.01	1
5	14	0.4	1000000	3	2	2	0.01	1
6	14	0.4	1000000	3	2	5	0.01	1

The results for this test case are summarized in Table 6. The best F-score was lower than that of the synthetic data set, however still significantly better than the random classifier (P = 0.033). The best run was determined to be run number 4 with an F-score of 40.13%. However run number 2 achieved the highest precision, with one replicate having 100% precision. The other replicates of this run were approximately 40% while run number 4 consistently had *>*80% precision. The best recall for a single replicate was 33.63% from run number 3. Again this was only one replicate, while run number 4 was more consistent with *>*20% recall. Even when considering the differences in baseline scores, run number 4 remained the best run. Again, the individual replicates are not shown here but are provided in [27].

**Table 6:** Average F-scores across replicates for each combination of parameters from Table 5 for the *Trypanosoma brucei *data set.

	Observed			Baseline			
**Run**	**Precision**	**Recall**	**F-Score**	**Precision**	**Recall**	**F-Score**	**F-Score Diff**

1	6.85%	0.33%	0.63%	0.18%	0.01%	0.01%	0.61%

2	46.59%	11.36%	18.26%	0.21%	0.07%	0.11%	18.15%

3	80.52%	15.82%	26.45%	1.15%	0.20%	0.33%	26.11%

4	**84.80%**	**26.29%**	**40.13%**	1.26%	0.38%	0.59%	**39.55%**

5	2.63%	5.84%	3.63%	2.78%	6.06%	3.82%	-0.19%

6	7.48%	21.13%	11.05%	2.89%	8.70%	4.34%	6.71%

Avg.	38.14%	13.46%	16.69%	1.41%	2.57%	1.53%	15.16%

Bold values are the best value for the category.

The best result of run number 4 produced 11 patterns shown in Table 7. The two most significant patterns are anchors flanked by stem-loops both downstream and upstream. There is a fair amount of overlap in the downstream stem-loop with the anchor. The elements that were expected are present but not in the order that was expected. The next two patterns are anchors broken into separate nodes either by gaps or areas of high GU%. The fifth pattern is the most interesting. It appears to not be the correct pattern, but if it is separated into two subgraphs it would produce two |0|1|0*| *patterns which is a correct anchor with an encompassing stem-loop. The gRNA seems to be able to edit at two different sites on the ATPase subunit 6 gene.

**Table 7:** Patterns produced by best replicate of run number 4.

P-value	**Freq**.	Pattern	TP%
9.25E-07	57	0|4|0|-|0|0|1|2|2|-|intra|inter|intra|	100%

2.92E-05	59	0|3|0|-|0|0|1|2|2|-|intra|inter|intra|	91.53%

2.18E-04	19	0|0|2|-|0|1|2|2|1|0|-|inter|inter|inter|	100%

4.47E-04	16	0|4|0|-|0|1|2|0|1|2|-|inter|inter|inter|	75%

7.62E-04	14	0|0|4|-|0|1|2|0|1|2|-|intra|inter|inter|	71.43%

0.001375	60	4|0|-|0|1|1|-|inter|intra|	96.67%

0.001895	11	0|3|0|-|0|1|2|2|0|1|-|inter|inter|intra|	54.55%

0.00224	21	0|0|3|-|0|1|0|2|1|-|intra|intra|inter|	80.95%

0.003987	169	0|3|-|0|1|0|-|intra|inter|	80.47%

0.005346	57	0|2|0|-|0|0|1|2|2|-|intra|inter|intra|	75.44%

0.005664	8	0|4|-|0|1|0|1|-|inter|inter|	37.50%

All of the embeddings for the first pattern correspond to the same anchor on the gene cytochrome oxidase subunit 3 and second pattern to NADH dehydrogenase subunit 7. Each embedding links the gene to gRNA gene on different minicircles. These are likely duplicate minicircles, since *Trypanosoma brucei *has many copies of each minicircle. As would be expected, the probability that these minicircle duplicates would occur by chance in the background data is very unlikely so this pattern would be drawn out. These minicircles are amongst those with the largest number of duplicates.

### Stacking energy experiment results

The *Trypanosoma brucei *data was used for additional experiments using a stacking energy model for filtering stems. The best parameters from the previous runs were used with an additional free energy (FE) parameter. The range for energy cutoffs chosen was from -12.00 kcal/mol to -16.00 kcal/mol. Outside of this range the number of nodes was either too large or too small to produce good results. Similarly to the previous runs, 6 replicates were used for each choice of energy cutoff. The results are summarized in Table 8.

**Table 8:** Results for experiments using stacking energy cutoff.

	Observed			Baseline			
**FE (kcal/mol)**	**Precision**	**Recall**	**F-score**	**Precision**	**Recall**	**F-score**	**F-score Diff**

-12	36.04%	17.16%	22.88%	0.64%	0.32%	0.42%	22.46%

-13	25.28%	20.88%	22.11%	1.33%	1.42%	1.31%	20.80%

-14	**30.98%**	**26.37%**	**28.16%**	1.87%	98.06%	1.95%	**26.22%**

-15	23.54%	34.31%	27.82%	3.88%	5.77%	4.62%	23.19%

-16	25.21%	30.11%	27.15%	5.51%	6.93%	6.14%	21.02%

The F-scores produced were consistently lower than the basic method. However this may be because the "best" parameters for the basic method are suboptimal for the free energy method. Specific replicates were able to achieve F-scores approximately equal to those produced from the basic method (data not shown). The patterns produced from one such replicate is shown in Table 9. Although the F-score is similar, the patterns produced are closer to the true structures. Nearly every pattern contains an anchor and a stem-loop, either downstream of, or encompassing, the anchor. The second pattern was examined more closely by comparing the positions of the stems to those in the KISS database. The instances where the anchor matched correctly, the stem-loop also matched those annotated in the database. This was not often seen in the results of the basic approach.

**Table 9:** Top ten patterns produced from the best replicate using stacking energy cutoff.

P-Value	Freq	Pattern	TP%
2.98E-09	112	4|0|-|0|1|1|-|true|false|-|	97.32%

2.86E-08	81	1|3|-|0|1|0|-|false|true|-|	58.03%

3.12E-07	56	4|0|0|-|0|1|1|0|2|2|-|true|false|false|-|	100%

4.99E-06	35	0|4|-|0|1|0|-|false|true|-|	91.43%

1.31E-04	222	0|3|-|0|1|0|-|false|true|-|	82.88%

2.55E-04	16	0|4|-|0|1|0|1|-|true|true|-|	75%

2.55E-04	80	3|0|-|0|1|1|-|true|false|-|	78.75%

3.39E-04	15	0|4|0|-|0|1|0|2|1|2|-|false|true|false|-|	100%

6.28E-04	26	0|2|0|-|0|1|2|2|1|0|-|true|true|false|-|	69.23%

6.28E-04	13	0|0|0|-|0|1|2|2|1|0|-|false|false|false|-|	0%

### Diplonema papillatum data

Some preliminary experiments were run on a third dataset with the mitochondrial genome from *Diplonema papillatum*. In this case the mechanism in unknown so precision and recall cannot be calculated. The hypothesized mechanism involves a RNA guide joining two mRNA modules by binding to 6nt regions at the ends of each module. Since the hypothesized anchors are of 6nt, the minimum complementary length was set to this value with a low 10% GU maximum to ensure only good anchors are created. The maximum number of molecules was increased to 3, since 3 are involved in the hypothetical mechanism. Normalization was set to 4 bins, and p-value and support were set to 0.1 and 1 respectively. The value of *K *was initially set to 3 to return the simplest possible structure, but the resulting set was empty. Increasing the *K *value to 4 returned many more results.

The top 5 results of this experiment are presented in Table 10. As can be seen in the pattern column of the table, 4 of the 5 patterns have two intermolecular nodes so they have potential to be a ppRNA. However, the patterns with two intermolecular nodes adjacent each other are always on the same module, which leaves patterns 1 and 3 as potential ppRNA. The first pattern has embeddings that include anchors on module 3/4 as well as anchors on module 7/8. The embeddings of the third pattern also cover module 3/4 and additionally module 4/5 and module 6/7.

**Table 10:** Top 5 results for *Diplonema papillatum *data.

P-Value	**Freq**.	Pattern
1.73E-06	12	0|0|0|0|-|0|1|2|2|1|3|-|inter|intra|intra|inter|

7.76E-06	20	0|0|0|0|-|0|1|2|3|2|0|1|3|-|inter|inter|intra|intra|

1.77E-05	9	0|0|0|0|-|0|1|2|1|3|3|-|inter|intra|inter|intra|

1.77E-05	9	0|0|0|-|0|1|2|0|1|2|-|intra|intra|intra|

5.81E-05	23	0|0|0|0|-|0|1|2|3|1|2|3|0|-|inter|inter|intra|intra|

**Figure 4:**
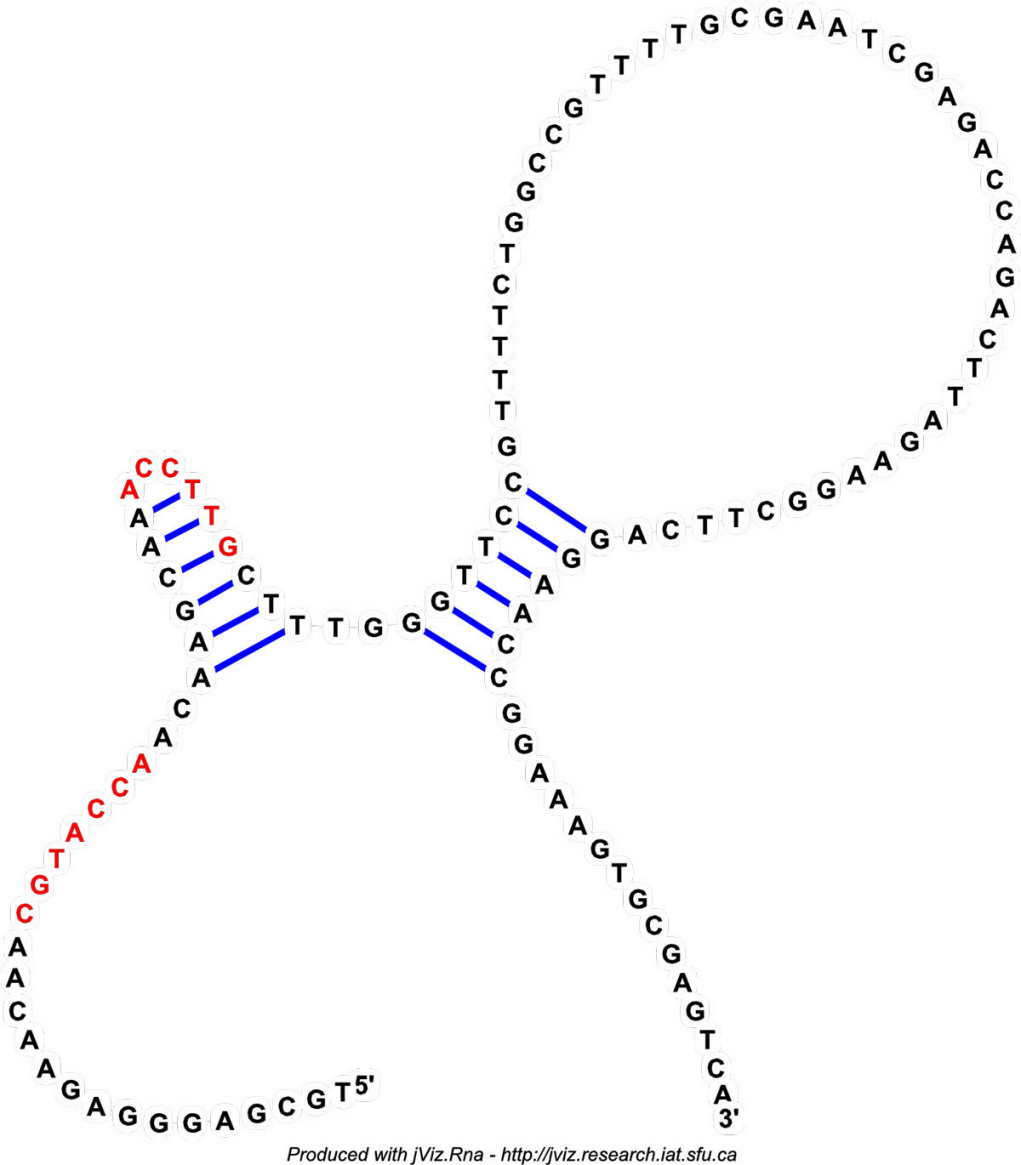
**Predicted ppRNA**. Predicted ppRNA based on the second pattern of the result set. The red regions mark the anchor which bind to module 4 and module 5.

From the third pattern, a hypothetical ppRNA can be constructed (Figure 4). This structure has two stems, one followed by the other, and two anchors near the first stem. The first anchor is just upstream of the stem, while the other one is within the stem. The double stem-loop with an anchor within the stem is similar to the gRNA structure. However the locations of the anchors on the modules are not near the end, so this may make this structure not viable.

### Performance comparison

There does not seem to be any published algorithms attempting to find both intermolecular and intramolecular structures on this scale. However there are many tools available for finding intramolecular, RNA structural motifs. Therefore for comparison we conducted experiments limiting the algorithm to find only intramolecular structures. The results were compared to those of the popular tool CMFinder 2.0 [28]. The dataset used to for these experiments was made up of 50 sequences, each approximately 270nt long, containing Selenocysteine insertion sequence 1 (SECIS1). This RNA structure contains 3 stems of various length nested within each other. The parameters used for RiboFSM allowed for stems of size 3 with free energy below -6 kcal/mol. No normalization was used and 1 million iterations were run. Furthermore the maximum mismatch parameter was used (set to 1), which was omitted in the previous experiments. This is because when considering only intramolecular stems the number of nodes is relatively small, so the algorithm can handle the additional imperfect matches. Default parameters were used for CMFinder but the tool returned no results. To resolve this the length of the motif candidates was restricted to 60-80nt long, since all the structures are within this range.

**Figure 5:**
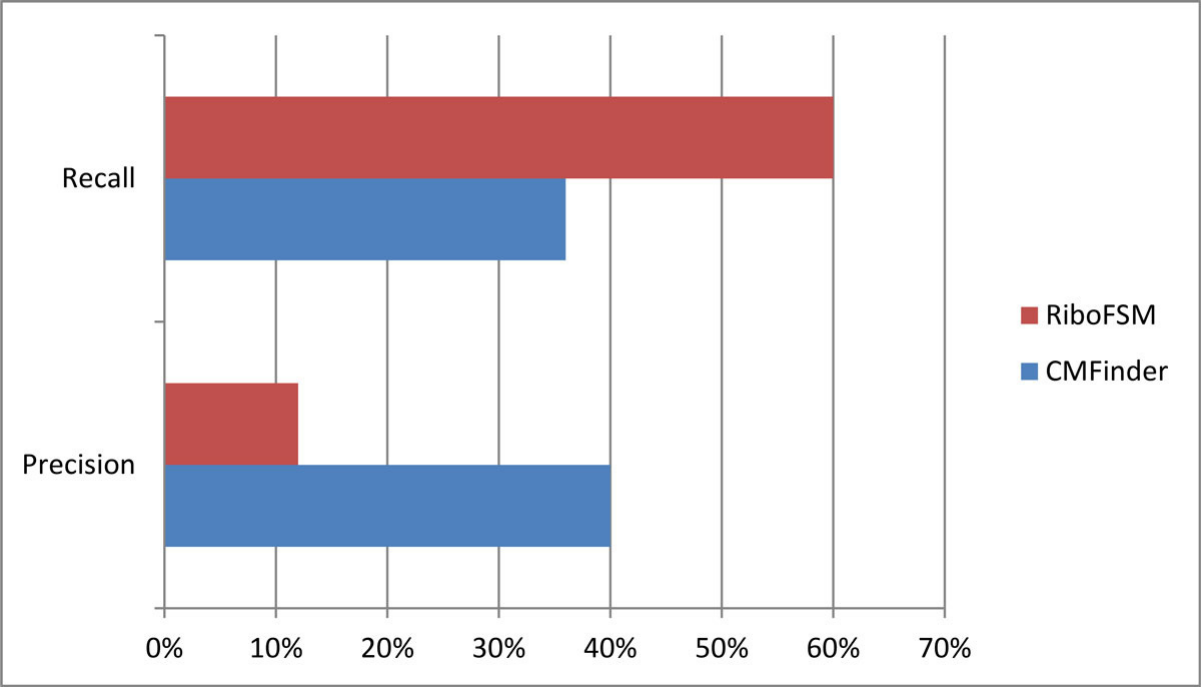
**Performance comparison**. Precision and recall for RiboFSM and CMFinder for the SECIS1 dataset.

The resulting precision and recall for RiboFSM was 60% and 12% respectively. For CMFinder, the precision was 40% and the recall was 36% (Figure 5). RiboFSM performed better than CMFinder in terms of recall but was worse in terms of precision. This is likely due to the size of the smallest stem, which is 3 nucleotides long. Setting the parameters to allow for nodes corresponding to such short stems, along with one mismatch, creates a large amount of noise. The p-value calculations, and subsequently the pattern filtering, is sensitive to noisy data. However RiboFSM was not designed to find such specific structures, but rather general patterns in sequences.

## Conclusion

We presented a novel framework for the discovery of RNA elements. It is an adaptation of frequent subgraph mining techniques on a large directed dual graph, representing all possible complementary sequences. This is the first instance of dual graphs being used in this way and the first application of frequent subgraph mining for this problem. Furthermore the dual graph representation was extended to not only represent secondary structures but also interactions between RNA.

It was applied to a synthetic and real-world data set, the *Trypanosoma brucei *mitochondrial genome, containing known gRNA and their target mRNA. The results are promising with the application being able to find patterns representing gRNA structures with high precision and recall. Furthermore the patterns linked these structures to their target mRNA. The algorithm was also applied to a *Diplonema papillatum *data set where the mechanism is unknown. RiboFSM was able to find patterns that could hypothetically guide editing in the organism. These results indicate that the algorithm will be able to predict novel gRNA and could later be applied to other RNA-related applications.

One limitation of RiboFSM is scalability in terms of graph building. Since the graphs being produced contain every possible complementary sequence, as the sequence length increases the number of nodes increases dramatically. Although the execution time remains reasonable the memory usage becomes very high, beyond the capabilities of an average desktop computer. This can be mitigated by increasing the minimum length of complementary regions, but this reduces recall and may yield the algorithm ineffective for finding structures with short stems. However the frequent subgraph mining component scales well as the graph size increases and is capable of running billions of iterations if necessary.

The algorithm can be improved or extended in a variety of ways. Currently the graph building component is the bottleneck in terms of runtime due to the naive approach used. Sophisticated methods exist for finding all complementary sequences which run more efficiently than the naive algorithm. One such method is the use of a generalized suffix tree or array as used in Seed [29]. This would make it possible to find all complimentary regions in linear time, improving on the current *O*(*n*^2^*k*) runtime. Furthermore, more heuristics could be developed to produce nodes which are most likely to be part of a real structure or mechanism using biological knowledge. For example, masking of low complexity regions.

Another way the algorithm may be extended is by investigating other sampling approaches. Many such approaches exist and have been used in other applications. One example would be Random Walks, where a root node is chosen and then the subgraph is extended by randomly choosing an edge. Probabilistic models could also be integrated at this point, where the probability of adding a node to the subgraph depends on properties of the node or how the node would influence the structure. This may be based on free energy or other properties of the complementary regions.

The labeling approach used can also be revisited. Many different combinations of labels containing a variety of information could be used. Currently the nodes are only labeled based on graphical properties. However labels can contain biological information as well, such as nucleotide composition or free energy. This process could also be more sophisticated, with labeling based on more than one node. This would be the case if a certain protein binding motif was known, which is very important since proteins are often involved with RNA mechanisms. The motif would always produce a specific subgraph, so if this subgraph was detected it could be labeled based on this information. This would allow for the creation of complex structures and systems. Also the annotations would be useful for interpreting the results.

The final area that can be explored is the calculation of statistical significance. Currently the distributions of ratios is assumed to be normal even though it is not quite normal. Different distributions can be explore to see if they are a better fit for the data, such as an extreme value distribution. The entire approach is fairly simple and could be replaced with a more complex statistical model. It would be especially effective if a model could be created that would not rely on background data at all. This would greatly reduce the runtime and memory usage of the algorithm. This could be done by predicting the properties of a randomized graph rather than producing the graph itself.

## Competing interests

The authors declare that they have no competing interests.

## Authors' contributions

ARG designed, implemented and tested RiboFSM under the supervision of MT. ARG wrote the initial draft of the manuscript. Both authors read and approved the final manuscript.
